# Synergistic immunomodulatory effect of synbiotics pre- and postoperative resection of pancreatic ductal adenocarcinoma: a randomized controlled study

**DOI:** 10.1007/s00262-024-03686-6

**Published:** 2024-04-25

**Authors:** Sara Maher, Hesham A. Elmeligy, Tarek Aboushousha, Noha Said Helal, Yousra Ossama, Mahmoud Rady, Ahmed Mohamed A. Hassan, Manal Kamel

**Affiliations:** 1 Lecturer of Immunology, Immunology Department, Theodor Bilharz Research Institute, Giza, Egypt; 2 Associate Professor of General Surgery, Surgery Department, Theodor Bilharz Research Institute, Giza, Egypt; 3 Professor of Pathology, Pathology Department, Theodor Bilharz Research Institute, Giza, Egypt; 4 Associate Professor of Pathology, Pathology Department, Theodor Bilharz Research Institute, Giza, Egypt; 5 Lecturer of Pathology, Pathology Department, October 6 University, Giza, Egypt; 6 Lecturer of Surgery, Department of Surgery, Theodor Bilharz Research Institute, Giza, Egypt; 7 Professor of Surgery, Surgery Department, Theodor Bilharz Research Institute, Giza, Egypt; 8 Professor of Immunology, Immunology Department, Theodor Bilharz Research Institute, Giza, Egypt

**Keywords:** Pancreatic ductal adenocarcinoma (PDAC), Probiotics, Synbiotics, CD8 + T cells

## Abstract

Pancreatic ductal adenocarcinoma (PDAC) is an aggressive cancer with a highly immunosuppressive microenvironment. This single-blind, randomized study aimed to evaluate the synergistic immunomodulatory effects of synbiotics (probiotics and inulin prebiotics), as well as their impact on postoperative complications and outcomes, compared to the use of probiotics alone. Ninety patients diagnosed with PDAC were enrolled and randomly assigned into three groups: the placebo group, the probiotics group (receiving a mixture of ten strains of Lactobacillus, Bifidobacterium, and Streptococcus bacteria at a dose of 25 billion CFUs), and the synbiotics group (the same probiotics along with inulin prebiotics). The interventions were administered for 14 days before the surgery and continued for one month postoperatively. Tumor tissue infiltration of CD8 + T cells and the expression of IFN γ were assessed by immunohistochemistry (IHC). Inflammatory cytokines concentrations, including Il 1 B, IL 6, and IL 10, were evaluated as well by ELISA at various time points pre- and postoperative. Furthermore, patients were followed up after the surgery to assess postoperative short-term outcomes. Our results showed a significant elevation of CD8 + T cell proportion and IFN γ expression in the synbiotics group compared to the probiotics group (*p* = 0.049, *p* = 0.013, respectively). Inflammatory cytokines showed a significant gradual decrease in the synbiotics group compared to placebo and probiotics-treated groups (*p* = 0.000 for both). Administration of synbiotics and probiotics significantly decreased the rate of postoperative complications including anastomotic leakage, diarrhea, and abdominal distension (*p* = 0.032, *p* = 0.044, *p* = 0.042, respectively), with a remarkable reduction in bacteremia in the synbiotics group. These results revealed that this synbiotics formulation potentially enhances the immune response and reduces complications associated with surgery.

*Clinical trial identification*: NCT06199752 (27-12-2023)

**Supplementary Information:**

The online version contains supplementary material available at 10.1007/s00262-024-03686-6.

## Introduction

Pancreatic cancer (PC) is a highly aggressive malignant tumor that ranked as the fourth leading cause of cancer-related deaths in the Europe and USA [1]. Considering all stages combined, PC has the poorest survival rates among all types of cancer, with only an 11% survival rate. It is projected to become the second leading cause of death by the year 2030 [2]. In Egypt, pancreatic cancer caused 2,906 deaths in 2020, accounting for 0.54% of total deaths, and with a mortality rate that varies by province, with northern districts having approximately 2.85 times higher rates than southern districts. This variation may be attributed to exposure to different environmental factors, as the northern regions have the highest rates of soil and water pollution in the country [3].

Pancreatic ductal adenocarcinoma (PDAC) is the most common type of pancreatic cancer, accounting for nearly all cases of pancreatic malignancies [4]. PDAC is defined as a tumor microenvironment that is highly stromal and exhibits weak immunogenicity. This microenvironment facilitates tumor evolution and plays a role in the development of resistance to therapy [5]. Surgical resection is the most commonly used strategy for PDAC treatment followed by adjuvant therapy but with long-term limited effectiveness [6]. Hence, it is crucial to investigate novel therapeutic approaches and supportive treatments that can improve the quality of patients’ lives [7]. Recently, there has been a growing focus on exploring the association between pancreatic cancer and different aspects of the gut microbiota [8, 9]. Consequently, the manipulation of the gut microbiota and the restoration of its diversity and balance might have a substantial impact on the management of this disease [10].

According to the consensus statement by the International Scientific Association for Probiotics and Prebiotics (ISAPP), probiotics are defined as living microorganisms that confer health benefits when consumed in adequate quantities [11]. Within the field of cancer research, probiotics have demonstrated the ability to bolster the immune response by attracting diverse immune cells, regulating inflammation, enhancing the integrity of the gut barrier, and exerting direct anti-tumor effects. These effects are achieved through mechanisms such as the production of antimicrobial peptides and the inhibition of cancer cell proliferation [12, 13]. The majority of research investigating the potential benefits of probiotic consumption on pancreatic cancer has been conducted using animal models. However, further investigation is necessary to fully comprehend its inhibitory role in the progression of pancreatic cancer. This requires additional exploration through preclinical and clinical studies [14].

Prebiotics are substrates that selectively utilized by host microorganisms, conferring a health benefit, such as mannan oligosaccharides, conjugated linoleic acids, polyunsaturated fatty acids, oligosaccharides such as fructooligosaccharides, inulin, galactooligosaccharides [15]. Prebiotics have also been shown to play a beneficial role in reducing the risk of inflammation and exhibiting anti-tumor effects [16]. On the other hand, synbiotics encompass the combination of probiotics and prebiotics, providing a potential synergistic effect in modulating the gut microbiota and promoting overall health [17]. In the context of cancer therapy, synbiotics may exert their effects through mechanisms such as immunomodulation, augmentation of chemotherapeutic effectiveness, and mitigation of treatment-related side effects [18]. Recent studies and a meta-analysis have suggested that the inclusion of probiotic/synbiotics supplements is associated with a significant reduction in the risk of postoperative infectious complications in individuals who have undergone surgery [19]. However, further research is needed to validate these findings, as there may be publication bias and limitations in the quality of evidence that could influence the results [20].

The objective of this study was to assess the synergistic effect of synbiotics (combination of probiotics and prebiotics) compared to probiotics alone in providing a significant anti-tumor immunomodulation effect in PDAC patients, as well as their impact on postoperative complications and outcomes.

## Methods

### Study design and patients

This single-blind randomized control study was conducted on patients with PDAC who underwent pancreatoduodenectomy in the General Surgery Department of Theodor Bilharz Research Institute Hospital and private hospitals. The data of all patients, including laboratory and pathological data, were collected from the electronic medical records of the TBRI hospital. All cases underwent Ct pancreatic protocol and EUS (endoscopic ultrasound) to exclude local vascular invasion before surgery (the criteria of acceptance is abutment in case of SMA (superior mesenteric artery), also abutment and encasement less than 180 degree in case of SMV (superior mesenteric vein) without vascular invasion. The inclusion criteria included patients with primary PDAC, with complete pathological and follow-up data, without long-distance metastasis, and any treatments before the surgery. The exclusion criteria included the patients who suffered from other tumors or died from accidental death or other diseases, lack of pathological and follow-up data, long-distance metastasis before the surgery, recently infected with COVID 19 or vaccinated with COVID 19 vaccines. Once the informed consent was obtained, the included patients were randomized, using randomization software, into placebo, probiotic, and synbiotics receiving groups. All included patients were blinded for the intervention.

**Fig. 1:**
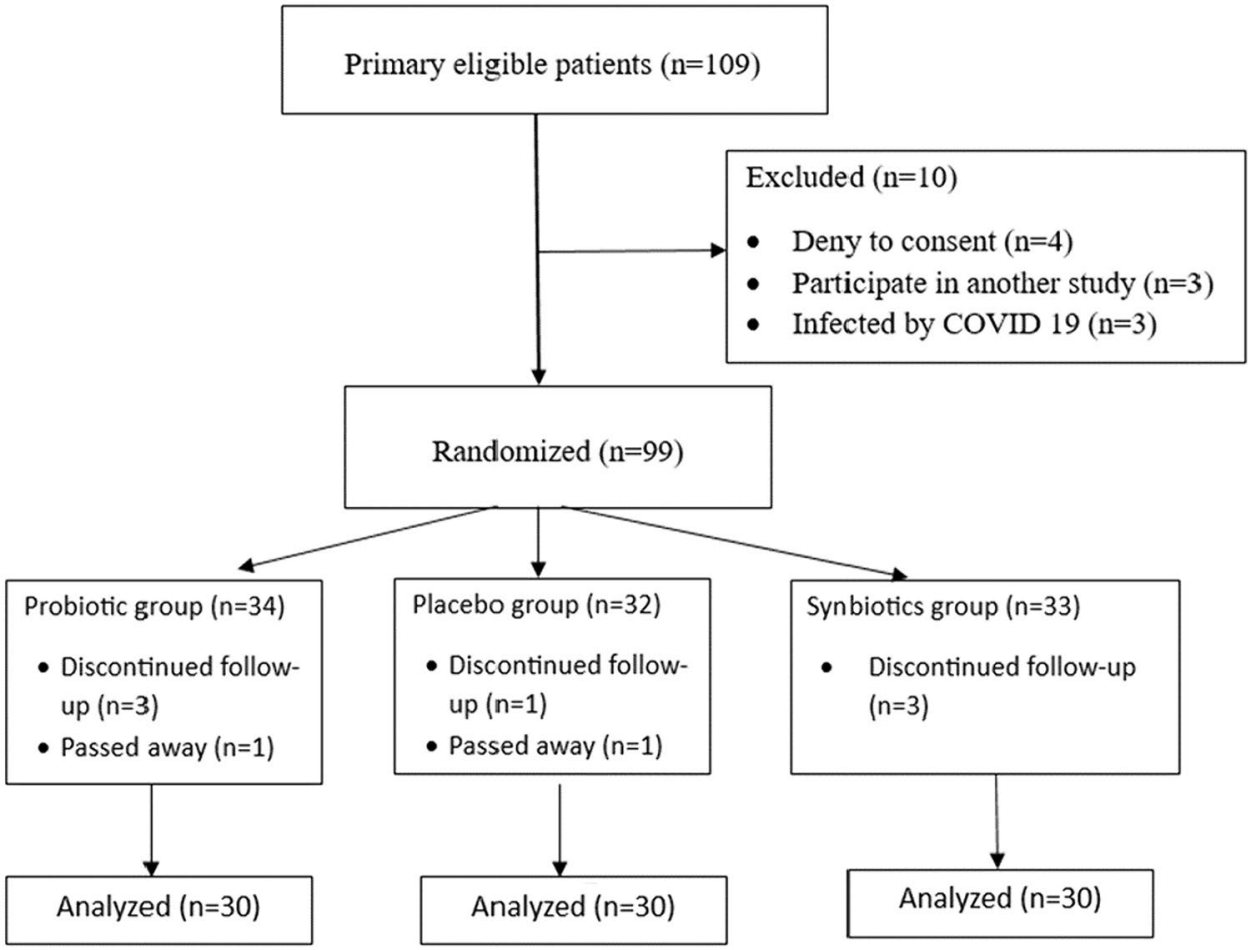
Consort diagram of study design

Between December 2021 and April 2023, and following the assessment of 115 primary eligible patients, 90 patients were randomly allocated into probiotics, synbiotics, and placebo groups (Fig. 1). Some patients were primarily excluded (*n = *10) because they denied consent (*n = *4), participating in another study (*n = *3) or were diagnosed by COVID 19 (*n = *3). Furthermore, during the follow-up period, we missed two patients in the placebo group, one of them due to discontinued follow-up, the other passed away one week postoperative due to pulmonary embolism inspired having anti-coagulant daily. In the probiotics group, we lost 2 cases, one for discontinued follow-up and the other passed away at home due to cardiac arrest post-angina one month postoperative. In the synbiotics group, we missed three patients due to discontinued follow-up.

### Probiotic and synbiotics treatment program

The probiotic and prebiotic medications utilized in this investigation are commercially available FDA-approved forms and have a well-established safety record. Throughout the administration process, both the patient and the nursing staff were obligated to report any possible side effects or unfavorable incidents. The study was halted if a patient withdrew their consent or if any significant adverse events related to the medication administration occurred. The probiotic supplement, 25 Billion CFU (Nowfoods, USA), composed of ten strains of bacteria (*Lactobacillus acidophilus, Bifidobacterium lactis, Lactobacillus plantarum, Lactobacillus paracasei, Bifidobacterium breve, Streptococcus thermophilus, Lactobacillus salivarius, and Bifidobacterium longum),* was taken in a dosage of two capsules once daily, two weeks (oral route) before the surgery and continued postoperatively for one month (first day postoperative by diluting the capsules in 50ml sterile water and given through feeding jejunostomy tube). For the synbiotic group, the same probiotic drug was used in addition to taking Inulin 1000 mg (Herbamama USA) two capsules once daily. For the placebo group, the same regimen was applied using a placebo drug.

### Blood samples collection

For all studied groups, blood samples were collected at the baseline, 14 days preoperative (Pre-14d), on the operation day (OP-0d), and 14 and 30 days postoperative (PO-14d and PO-30, respectively). Blood was collected into BD Vacutainer tubes and allowed to clot for 30 min. Afterward, it was centrifuged for 15 min at 1300 rpm. The separated serum was then stored in a −80°C freezer until analysis.

### Pancreaticoduodenectomy preparation and techniques

All patients were subjected to a carbohydrate-loading regimen before the Whipple operation. Fasting before operation was restricted only to 6 h for food and 2 h for fluids and also first day postoperative fluid was introduced through feeding jejunostomy to decrease the catabolic state postoperative. Eighty patients were operated by the open technique of Whipple and only ten patients were operated by the laparoscopic technique of Whipple and in all cases feeding jejunostomy are routinely added to facilitate early feeding. Pancreatojejunostomy end to side 2 layered ducts to the mucosa with the placing of the internal stent with omental wrapping was done in all cases. Hepaticojejunostomy was done as a posterior continuous parachuting technique and anterior interrupted suturing. Gastrojejunostomy (pylorus-preserving) was done by stapling side to side. The feeding jejunostomy tube was placed 40 cm distal to the gastrojejunostomy. Three drains were placed (Morrison, retrogastric, and pelvic) and closure in layers. For the Laparoscopic technique, we start with the true umbilical open technique for insufflation of the abdominal cavity by CO2 through a 10 mm port. Fife ports were introduced. All steps were similar to the open technique but the specimen was delivered through an incision in the left lumbar region.

### Histopathological and immunohistochemical techniques

Pancreatic samples were submitted to the Pathology Department, TBRI, for gross and microscopic examination. Pancreatic tumor tissues were processed for preparation of formalin-fixed paraffin-embedded blocks. Sections of 4 um thick were cut and spread on three positively charged glass slides. One of them was stained by Hematoxylin and Eosin (H&E) for evaluation of histopathological changes. The other two slides were underwent immunohistochemical procedure as incubated with anti-CD8 polyclonal antibody (YPA2235, Biospes, Chongqing, China), and anti- IFNγ polyclonal antibody (YPA2285, Biospes, Chongqing, China), at dilution of 1:100. Negative controls were carried out in which phosphate buffered saline was used instead of the primary antibody.

### Immunohistochemical interpretation and scoring

Scoring of CD8 and IFNγ immunostaining was performed blindly to the patients’ clinicopathological data. CD8 + T cells and IFNγ staining were interpreted as positive when > 10% of cells showed cytoplasmic or nuclear staining. The mean percentage of positive cells was calculated in 10 randomly selected fields at high magnification (× 400). Interpretation of stained slides was done using a light microscope (Scope A1, Axio, Zeiss, Germany). Photomicrographs were taken using a microscope camera (AxioCam, MRc5, Zeiss, Germany).

### Enzyme-linked immunosorbent assay (ELISA)

Serum concentrations of IL-10, IL-6, and IL-1β were determined using sandwich-based enzyme-linked immunosorbent assay (ELISA) kits, following the manufacturer’s instructions (Sunlong Biotech, China). Briefly, 50 μL of diluted samples or standards were incubated in a micro-ELISA strip plate that was pre-coated with antibodies specific to the tested cytokines. Horseradish peroxidase (HRP)-Conjugate was employed as a detection antibody. After incubation for 30 min at 37°C and three subsequent washes, TMP substrate was used to visualize the HRP-enzymatic reaction. The absorbance of the produced color was measured at 450 nm using an ELISA reader (Multiscan TMFC, Thermofisher, USA), and the cytokine concentration was then calculated from the standard curve.

## Statistical analysis

Sample size was calculated using G*Power program (University of Düsseldorf, Düsseldorf, Germany) to calculate difference between mean of two independent groups. The included patients were randomized, using Excel RAND function. Data were analyzed using statistical software package (IBM-SPSS) version 23 software. Kolmogorov–Smirnov test showed that the raw data were normally distributed. One ANOVA was applied to study the effect of treatment on the studied parameters. Two-way ANOVA was applied to study the effect of time and treatment on the studied parameters. The least significant difference (LSD) test was used to illustrate the statistical differences among the experimental groups. Duncan’s test was used to illustrate the homogeneity among the different intervals (Table 1).

**Table 1:** Demographic characteristics of participants

Variable		Placebo N (%)	Probiotics N (%)	Synbiotics N (%)	*p*-value
Age category (year)	< 50	17(56.7)	12 (40)	16 (53.3)	0.393
	> 50	13 (43.3)	18 (60)	14 (46.7)	
Gender	Male	21 (70)	17 (56.7)	24 (80)	0.147
	Female	9 (30)	13 (43.3)	6 (20)	
Disease history	None	25 (83)	27 (90)	26 (86)	0.210
	Diabetes	2 (7)	1 (3)	2 (7)	
	Hypertension	3 (10)	2 (7)	2 (7)	
BMI (Mean ± SE)		22.40 ± 1.85	23.00 ± 1.27	22.00 ± 1.96	0.919

Data are displayed as mean ± standard error of the mean

*p* > 0.05: represents an insignificant effect

BMI: body mass index

### Tissue infiltration of CD8 + T cells and IFN-γ expression

**Fig. 2:**
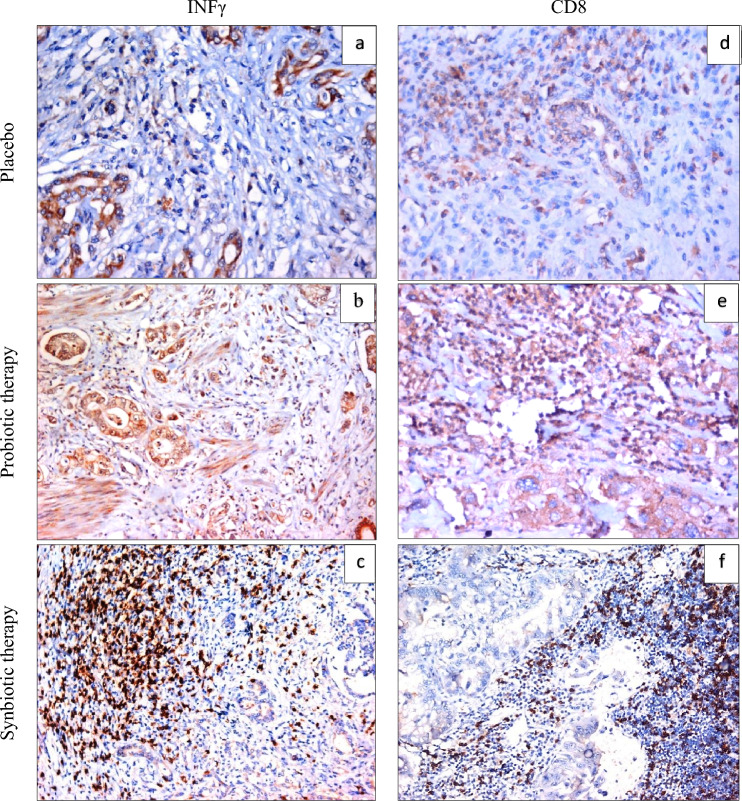
IHC expression of INFγ and CD8 in PDAC **a** PDAC, not receiving probiotic/synbiotic therapy, showing positive expression of INFγ in malignant glands and in < 10% of associated inflammatory cells, **b** PDAC, receiving preoperative probiotic therapy, showing positive expression of INFγ in malignant glands and in > 50% of associated inflammatory cells, **c** PDAC, receiving preoperative synbiotic therapy, showing positive expression of INFγ in > 60% of associated inflammatory cells (IHC × 200, **d** PDAC, not receiving probiotic/synbiotic therapy, showing positive expression of CD8 in < 10% of associated inflammatory cells, **e** PDAC, receiving preoperative probiotic therapy, showing positive expression of CD8 in > 50% of associated inflammatory cells, **f** PDAC, receiving preoperative synbiotic therapy, showing positive expression of CD8 in > 60% of associated inflammatory cells (IHC × 200). IHC; immunohistochemistry, PDAC; pancreatic ductal adenocarcinoma

Tumor tissue infiltration of CD8 + T cells as well as the expression of IFN γ in tissue samples from 90 patients with PDAC were assessed by IHC (Fig. 2(a)–(f)). It was observed that patients who received synbiotics and probiotics before surgery exhibited a significant increase in both the proportion of CD8 + T cells as well as the expression of IFN-γ compared to those in the placebo group (*p* = 0.000). Moreover, the expression of IFN-γ as well as CD8 + T cells infiltration was notably higher in the synbiotics-treated group in comparison to the probiotics-treated group (*p* = 0.013, *p* = 0.049, respectively) (Table 2).

**Table 2:** Tissue expression of CD8 + cells and INF-γ in subjected groups

Variable	Groups	Mean ± SE	*p*-values
INF-γ	Placebo	15.83 ± 2.28	–
	Probiotics	52.50 ± 5.66 *	P_1_ = 0.002
	Synbiotics	60.50 ± 6.03*	P_1_ = 0.000, P_2_ = 0.013
CD8	Placebo	15.83 ± 2.63	–
	Probiotics	54.83 ± 8.32*	P_1_ = 0.005
	Synbiotics	61.50 ± 6.62*	P_1_ = 0.002, P_2_ = 0.049

* represents significant differences (P_1_ < 0.05), as compared to the placebo group

# represents significant differences (P_2_ < 0.05), as compared to the probiotics group

### Association between clinicopathological parameters and tissue expression of INF-γ and CD8 + T cells

On studying the association between the expression of INFγ and CD8 infiltration in the tumor tissues, and the clinicopathological parameters in the preoperative studied groups, we observed that pancreatic tissue exhibited a significant upregulation in the expression of INF-γ and CD8 + T cells infiltration in both synbiotics and prebiotics treated groups compared to the placebo group. Moreover, as compared to the probiotic group, significant increases in the expression of INFγ and CD8 in the synbiotic group were obtained. In probiotics-treated group, a significant elevation in the expression of INFγ was observed in female patients (*p* = 0.011) younger than 50 years old compared to males, while in probiotics and synbiotics groups there were significant elevations in the expression of INF-γ with the pN-stage (*p* = 0.002, *p* = 0.025) as well as TNM stage (*p* = 0.000, *p* = 0.025), respectively. In the synbiotic group, the significantly (*p* = 0.036) higher pN-stage as well as TNM stage is proportional to the greater expression of CD8 (Table 3).

**Table 3:** The clinicopathological parameters on the expression of INFγ and CD8 + T cells in the tumor tissues of all groups

Clinicopathological parameters		INFγ			CD8		
		Placebo	Probiotic	Synbiotic	Placebo	Probiotic	Synbiotic
Age category	< 50	13.19 ± 0.74	27.50 ± 2.26*	35.89 ± 1.76*#	23.88 ± 0.77	32.25 ± 1.56*	42.78 ± 2.40*#
	*p* value	–	P_2_ = 0.000	P_2_ = 0.000, P3 = 0.001	–	P_2_ = 0.000	P_2_ = 0.000, P3 = 0.000
	> 50	13.65 ± 0.90	26.47 ± 2.01*	36.75 ± 1.34*#	26.15 ± 0.96	31.40 ± 1.30*	46.50 ± 1.50*#
	*p* value	–	P_2_ = 0.000	P_2_ = 0.000, P3 = 0.000	–	P_2_ = 0.005	P_2_ = 0.000, P3 = 0.000
	*p* value	P_1_ = 0.703	P_1_ = 0.735	P_1_ = 0.714	P_1_ = 0.083	P_1_ = 0.677	P_1_ = 0.188
Gender	Male	12.43 ± 0.55	23.43 ± 1.97*	36.60 ± 1.56*#	23.48 ± 0.76	32.29 ± 1.34*	46.60 ± 2.06*#
	*p* value	–	P_2_ = 0.000	P_2_ = 0.000, P3 = 0.000	–	P_2_ = 0.000	P_2_ = 0.000, P3 = 0.000
	Female	14.87 ± 1.11	30.69 ± 1.74*¶	36.42 ± 1.42*#	27.47 ± 0.85¶	31.23 ± 1.49	44.68 ± 1.67*#
	*p* value	–	P_2_ = 0.000	P_2_ = 0.000, P3 = 0.004	–	P_2_ = 0.064	P_2_ = 0.000, P3 = 0.000
	*p* value	P_1_ = 0.063	P_1_ = 0.011	P_1_ = 0.939	P_1_ = 0.001	P_1_ = 0.602	P_1_ = 0.491
pT-stage	pT2	12.50 ± 0.61	27.50 ± 1.88*	35.92 ± 1.75*#	23.00 ± 0.70	33.57 ± 1.02*	46.77 ± 1.82*#
	*p* value	–	P_2_ = 0.000	P_2_ = 0.000, P3 = 0.000	–	P_2_ = 0.000	P_2_ = 0.000, P3 = 0.000
	pT3	14.05 ± 0.87	26.31 ± 2.37*	36.94 ± 1.33*#	26.50 ± 0.86	29.85 ± 1.60	44.19 ± 1.82*#
	*p* value	–	P_2_ = 0.000	P_2_ = 0.000, P3 = 0.000	–	P_2_ = 0.069	P_2_ = 0.000, P3 = 0.000
	*p* value	P_1_ = 0.206	P_1_ = 0.695	P_1_ = 0.642	P_1_ = 0.007	P_1_ = 0.057	P_1_ = 0.329
pN-stage	pN1	13.78 ± 0.75	21.73 ± 1.91*	34.07 ± 1.11*#	24.89 ± 0.90	33.45 ± 1.72*	42.57 ± 1.60*#
	*p* value	–	P_2_ = 0.000	P_2_ = 0.000, P3 = 0.000	–	P_2_ = 0.000	P_2_ = 0.000, P3 = 0.000
	pN2	13.11 ± 0.92	30.50 ± 1.62*¶	38.73 ± 1.58*#¶	25.39 ± 0.97	30.63 ± 1.12*	47.93 ± 1.80*#¶
	*p* value	–	P_2_ = 0.000	P_2_ = 0.000, P3 = 0.000	–	P_2_ = 0.004	P_2_ = 0.000, P3 = 0.000
	*p* value	P_1_ = 0.58	P_1_ = 0.002	P_1_ = 0.025	P_1_ = 0.707	P_1_ = 0.162	P_1_ = 0.036
TNM stage	Stage II	13.78 ± 0.75	20.60 ± 1.71*	34.07 ± 1.11*#	24.89 ± 0.90	32.90 ± 1.80*	42.57 ± 1.60*#
	*p* value	–	P_2_ = 0.001	P_2_ = 0.000, P3 = 0.000	–	P_2_ = 0.000	P_2_ = 0.000, P3 = 0.000
	Stage III	13.11 ± 0.92	30.65 ± 1.53*¶	38.73 ± 1.58*#¶	25.39 ± 0.97	31.12 ± 1.16*	47.93 ± 1.80*#¶
	*p* value	–	P_2_ = 0.000	P_2_ = 0.000, P3 = 0.000	–	P_2_ = 0.002	P_2_ = 0.000, P3 = 0.000
	*p* value	P_1_ = 0.58	P_1_ = 0.000	P_1_ = 0.025	P_1_ = 0.707	P_1_ = 0.393	P_1_ = 0.036

pT-stage; pathological tumor stage, pN-stage; pathological lymph node stage, TNM: tumor lymph nodes metastasis stage. Data are displayed as mean ± standard error

*, #: significant differences as compared to the placebo (P_2_ < 0.05) and probiotic (P3 < 0.05) groups. In each group, ¶: significant differences (P_1_ < 0.05), as compared to values at age < 50 or Males or pT2 or pN1 or Stage

### Serum concentrations of IL-1 β, IL 6, and IL 10

**Fig. 3:**
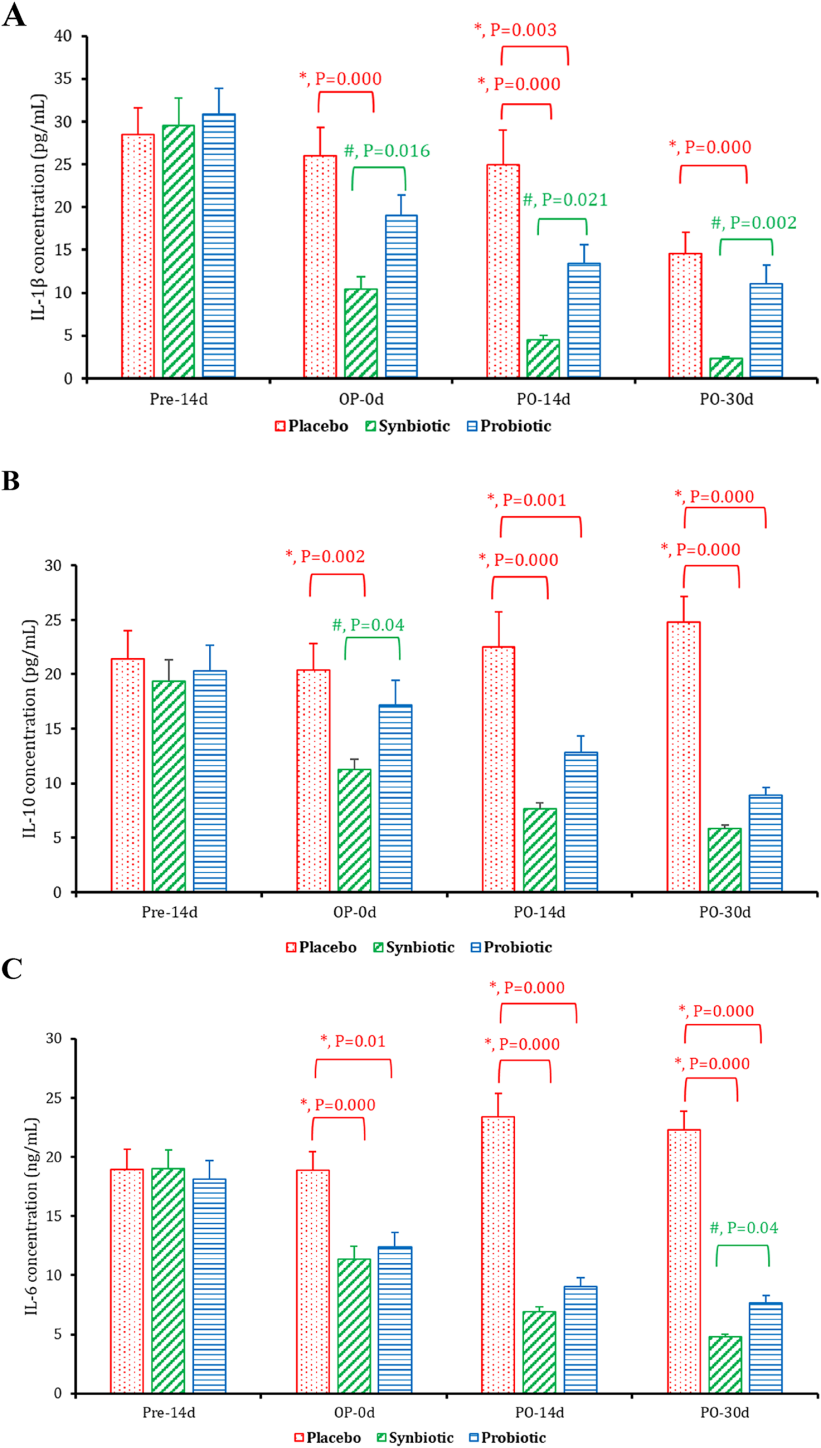
The concentrations of serum cytokines (IL-1B (**A**), IL-10 (**B**), and IL-6 (**C**)) measured in the placebo, probiotics, and synbiotics-treated groups at different time intervals: 14 days before the surgery (Pre-14d), on the surgery date (OP-0d), 14 days postoperative (PO-14d), and thirty days postoperative (PO-30d). Data is displayed as mean ± standard error. *: a significant difference (P_1_ < 0.05), as compared to the placebo group. #: a significant difference (P_2_ < 0.05), as compared to the symbiotic group

Cytokine concentrations at different time points pre- and post-tumor resection were evaluated for all subjected groups, 14 days before surgery (Pre-14D), at the operation day (OP-0d), 14 and 30 days postoperative (PO-14d and PO-30d, respectively). In the placebo group, there was no significant difference in the IL-1β concentrations at different time points but at PO-30d, where a significant decline was recorded, as compared to the baseline. In addition, as compared to the placebo group, a significant decline was observed at OP-0d, PO-14, and PO-30d (*p* = 0.000) in the synbiotics-treated group and at PO-14 and PO-30d in the probiotics-treated group (*p* = 0.000). However, the IL-1 β concentrations showed a significant decline in the synbiotics group compared with the probiotics group at the OP-0d (*p* = 0.016), PO-14d (*p* = 0.021), and PO-30 (*p* = 0.002) (Table 4, Fig. 3A). For IL-10 assessment, in the placebo group, there was no significant difference in the IL-10 concentrations among all the time intervals. In the synbiotics group and probiotics group, a gradual reduction in the IL-10 concentrations was observed from the OP-0d to PO-30d, as compared to the baseline (*p* = 0.002, *p* = 0.000, *p* = 0.000, respectively). Moreover, as compared to the placebo group, on the PO-14d and PO-30d, the IL-10 concentrations significantly declined (*p* = 0.000). However, as compared to the probiotics group, the IL-10 concentrations showed a significant decline at OP-0d (*p* = 0.04) (Table 4, Fig. 3B). Regarding IL-6 concentration, there was no significant difference among all time intervals. In the probiotic and synbiotics group, a gradual reduction was detected from the OP-0d to PO-30d, as compared to the baseline. In addition, as compared to the placebo, on the OP-0d, PO-14d, and PO-30d, the IL-6 concentrations significantly declined (*p* = 0.000) in both groups (*p* = 0.000). However, as compared to the probiotic group, the IL-6 concentrations showed a significant decline at PO-14d (*p* = 0.039) (Table 4, Fig. 3C).

### Postoperative short-term outcomes

Postoperative short-term outcome conditions were evaluated in all groups included in the study (Table 5). Both probiotics and symbiotics groups demonstrated a significant reduction in the average number of days until the first bowel movement, in comparison to the placebo group (*p* = 0.000). There were no significant differences in the postoperative hospital stay period as well as the days till the return to normal activity. Incidence of infectious complications showed significant improvement in bacteremia in probiotics and synbiotics groups (0.047) with a remarkable decrease in the synbiotics group as well as the incidence of pneumonia. Non-infectious complications, including anastomotic leakage, diarrhea, and abdominal distension, showed significant improvement in synbiotics and probiotics receiving groups compared to the placebo group (*p* = 0.032, *p* = 0.044, and *p* = 0.42, respectively). Moreover, diarrhea and abdominal distension were particularly noteworthy decreases in the synbiotics group. Three cases with anastomotic leakage in placebo group that did not need reoperation including 2 cases with pancreaticojejunostomy leakage which were treated conservatively and closed within 3 weeks and only one case of biliary leakage which was treated by internal external stent placed for one month.

## Results

### Demographic characteristics of the studied patients

The studied groups demonstrated homogeneity in their baseline features. The results indicated that there were no significant differences among the groups in terms of demographic characteristics, including sex, age, history of chronic disease, and body mass index (Table 1).

## Discussion

In contrast to the numerous studies conducted on colorectal cancer (CRC) and gastric cancer (GC), there is a lack of research exploring the role of synbiotics in the prevention and treatment of other gastrointestinal (GI) cancers, such as pancreatic and liver cancer. Only a limited number of published reports have investigated the potential link between probiotics and prebiotics in pancreatic ductal adenocarcinoma (PDAC), focusing primarily on the suppression of tumorigenesis and the impact on post-surgical complications [21, 22]. To the best of our knowledge, this is the first randomized clinical study to evaluate the immunomodulatory effect and post-surgical complications of oral synbiotics (probiotics and inulin) compared to probiotics alone in PDAC patients. Our results indicated that consuming synbiotics and probiotics supplements for six weeks (two weeks preoperative and four weeks postoperative), significantly improved the immune response in the treated groups compared to the placebo group. Immunohistochemical results showed that the proportion of CD8 + T cells and the expression of IFN-γ in tumor tissue were significantly higher in patients treated with synbiotics or probiotics compared to those treated with a placebo. Additionally, the synbiotics group showed a greater elevation in CD8 + T cells and IFN-γ expression compared to the probiotics group. These findings are consistent with the results obtained by Mao et al. [23], who suggested that probiotics could enhance the anti-tumor immune response of CD8 + T cells and IFN-γ + T cells in the tumor microenvironment in patients with colorectal carcinoma. In our study, we used a ten-strain cocktail of probiotics including *Bifidobacterium, Lactobacillus, and Streptococcus* bacteria strains in addition to inulin fibers prebiotics. Several studies have demonstrated the efficient immunomodulatory effects of such strains. For example, Yoon et al. [24] found that supplementation with *B. breve* strains reduced tumor growth in mice with colon carcinoma by augmenting lymphocyte-mediated anti-cancer immunity. Similarly, Lee et al. [25] concluded that *Lactobacillus acidophilus* strains increased serum IFN-γ, CD4 + , and CD8 + cells in mice with induced colon cancer. Other studies have also shown the modulation of the anti-tumor immune response by using a combination of *Bifidobacterium* or *Lactobacillus acidophilus* bacteria, leading to the improvement of dendritic cells function, infiltration of CD8 + T cells, and reduction of inflammatory cytokines [26, 27].

In our study, the significant increase in the expression of CD8 + T cells and IFN-γ in the synbiotics group compared to the probiotics group may be attributed to the beneficial effect of consuming inulin prebiotics along with these probiotic strains. These results are consistent with various preclinical studies that have suggested the anti-tumor immune system effect of inulin, particularly when combined with different probiotic regimens. For instance, Kassayová et al. [28] found that consuming *Lactobacillus plantarum* along with inulin prebiotics reduced carcinogenesis by inducing apoptosis and diminishing pro-inflammatory cytokines in a colon cancer model. Also, Li et al. (2020) used inulin and mucin prebiotics in their study on colon cancer in mice and observed that dietary consumption of inulin, but not mucin, reduced tumor growth through the induction of CD8 + T and CD4 + T cells mediated anti-tumor immune response [25]. Boucher et al. [29] also demonstrated that an inulin-enriched diet along with probiotic strains triggered an enhanced CD4 + and CD8 + αβ T cell-mediated anti-tumor response and attenuated tumor growth in preclinical tumor-bearing mouse models.

Elevated levels of inflammatory-mediated cytokines such as IL-6 and IL-10 have been associated with poor prognosis in patients with pancreatic cancer [30–32]. In our study, we evaluated the concentration of circulating inflammatory-mediated cytokines (IL-10, IL-6, and IL-1ß) in all groups at different time intervals before and after tumor resection. Our results showed a significant decrease in all tested cytokines in both the probiotics and synbiotics groups compared to the placebo group. The synbiotics group exhibited a more pronounced decline in IL-1ß levels on the day of the operation, 14 days, and 30 days after surgery. These findings are consistent with Das et al. [33], who concluded that neutralizing IL-1ß could promote intratumoral CD8 + T cell infiltration and function and sensitize pancreatic ductal adenocarcinoma to checkpoint immunotherapy. Additionally, we found that IL-6 and IL-10 levels gradually decreased on the day of the operation and 30 days postoperative compared to the baseline. The synbiotics group showed a significant reduction in IL-6 levels 14 days postoperative and in IL-10 levels on the day of the operation compared to the probiotics group. This reduction in cytokine levels can be attributed to the synergistic anti-inflammatory effect of synbiotics, particularly due to the inclusion of inulin fibers as a prebiotic. These results align with other studies that have discussed the anti-inflammatory and immunomodulatory effects of inulin prebiotics [34, 35].

Considering that surgery is the primary determinant of prognosis in PDAC, and holds curative potential, our study aimed to investigate the short-term postoperative outcomes when probiotics or synbiotics were administered. Limited research has been conducted on the supplementation of probiotics or synbiotics in patients undergoing pancreaticoduodenectomy. Between 2007 and 2021, only six studies with conflicting results were carried out, involving a total of 294 participants (147 in the control group and 147 in the intervention group). Three studies utilized probiotics with limited strains, Folwarski et al. [36], Diepenhorst et al. [37], and Nomura et al. [38], and three studies used synbiotics, including Yokoyama et al. [39], Sommacal et al. [40], and Rayes et al. [41]. In our study, we observed a significant reduction in the average number of days until the first bowel movement and the incidence of diarrhea in both the synbiotics and probiotics groups compared to the placebo group. The reduction was particularly notable in the synbiotics group. These findings are consistent with the results obtained by Folwarski et al. [36] and Diepenhorst et al. [37], who observed that the consumption of *Lactobacillus* strains improved bacterial translocation and postoperative outcomes after pancreatoduodenectomy. Furthermore, our results showed a decrease in infectious complications, in both the probiotics and synbiotics groups compared to the placebo group, with a more pronounced improvement observed in the synbiotics group. These findings are in line with the research conducted by Rayes et al. [41]. In their prospective randomized trial involving 80 patients who underwent pylorus-preserving pancreatoduodenectomy (PPPD) and received lactobacillus and fibers for 30 days, they observed a significant reduction in bacterial infection rates and anastomotic leakage following PPPD. Sommacal et al. [40] also concluded that perioperative synbiotics supplementation decreases postoperative complications in periampullary neoplasms. Their study included 23 patients who received a cocktail of Lactobacillus strains and fructooligosaccharides twice daily for 14 days, resulting in improved hospital stay length and a decreased incidence of infection. Regarding postoperative hospital stay and the time required for normal activities, there were no significant differences observed among the groups. We believe that this is directly related to our protocol in TBRI, which includes routine feeding jejunostomy in all cases and the use of a new technique for anastomosis, specifically end-to-side duct to mucosa with an internal stent and two-layered pancreaticojejunostomies with omental wrapping. Limitations of this study include the limited sample size resulting from the selectivity criteria applied to the included patients. Future research could benefit from broader investigations encompassing a wider range of parameters and longer observation periods, including the prognosis period. Additionally, deeper investigations into multiple additional factors could be conducted to enhance our understanding in subsequent studies.

## Conclusion

This study provided evidence of the significant immunomodulatory impact of synbiotics supplementation and its contribution to improving postoperative complications in PDAC patients. However, further large-scale studies are necessary to explore more pertinent clinical evidence in this field. These studies should involve diverse combinations and regimens of probiotics and prebiotics administered over extended periods. Additionally, investigating the potential synergies between synbiotics and immunotherapeutic drugs is crucial. By conducting such research, we can gain a deeper understanding of the topic and obtain more valuable clinical insights.

### Supplementary Information

Below is the link to the electronic supplementary material.

Supplementary file1 (DOCX 16 kb)

## Data Availability

All data generated or analyzed during this study are included in this manuscript.
