# Supplementary material for: Synergistic immunomodulatory effect of synbiotics pre- and postoperative resection of pancreatic ductal adenocarcinoma: a randomized controlled study
Source: Cancer Immunol Immunother. 2024 Apr 25;73(6):109. doi: 10.1007/s00262-024-03686-6 (PMC11045696; doi:10.1007/s00262-024-03686-6)
Supplement: Supplementary file 1 — Supplementary file1 (DOCX 16 kb) [file 262_2024_3686_MOESM1_ESM.docx]

**Table 4.** The concentrations of IL-1β, IL-10 and IL-6 in all the studied groups. Data is displayed as mean ± standard error.

|  | **Groups** | **Sampling time** | | | | **Effect of Time** |
| --- | --- | --- | --- | --- | --- | --- |
|  |  | **Pre-14d** | **OP-0d** | **PO-14d** | **PO-30d** |  |
| **IL-1B** | **Placebo** | 28.43 ± 3.15^b^ | 25.97 ± 3.31^b^ | 24.91 ± 4.08^b^ | 14.60 ± 2.51^a^ | F_3,116_=3.39, P=0.02 |
|  | **Synbiotics** | 29.49 ± 3.24^c^ | 10.44 ±1.44^b*^ | 4.47 ± 0.59^a*^ | 2.27 ± 0.23^a*^ | F_3,116_=47.22, P=0.000 |
|  |  | P1=0.813 | P1=0.000 | P1=0.000 | P1=0.000 |  |
|  | **Probiotic** | 30.88 ± 3.03^c^ | 19.06 ± 2.36^b#^ | 13.42 ± 2.16^a*#^ | 11.05 ± 2.21^a#^ | F_3,116_=12.89, P=0.000 |
|  |  | P1=0.583, P2=0.754 | P1=0.053, P2=0.016 | P1=0.003, P2=0.021 | P1=0.198, P2=0.002 |  |
|  | **Treatment** | F_2,87_=0.153, P=0.858 | F_2,87_=3.75, P=0.000 | F_2,87_=14.55, P=0.000 | F_2,87_=10.76, P=0.000 |  |
| **IL-10** | **Placebo** | 21.42 ± 2.56^a^ | 20.36 ± 2.44^a^ | 22.48 ± 3.22^a^ | 24.79 ± 2.36^a^ | F_3,116_=0.504, P1=0.681 |
|  | **Synbiotics** | 19.34 ± 1.98^c^ | 11.31 ± 0.94^b*^ | 7.69 ± 0.49^a*^ | 5.85 ± 0.29^a*^ | F_3,116_=27.96, P1=0.000 |
|  |  | P1=0.524 | P1=0.002 | P1=0.000 | P1=0.000 |  |
|  | **Probiotic** | 20.31 ± 2.32^c^ | 17.19 ± 2.26^b#^ | 12.81 ± 1.50^a*^ | 8.89 ± 0.77^a*^ | F_3,116_=7.48, P1=0.000 |
|  |  | P1=0.734, P2=0.765 | P1=0.265, P2=0.04 | P1=0.001, P2=0.084 | P1=0.000, P2=0.14 |  |
|  | **Treatment** | F_2,87_=0.205, P2=0.815 | F_2,87_=5.29, P2=0.007 | F_2,87_=13.14, P2=0.000 | F_2,87_=49.69, P2=0.000 |  |
| **IL-6** | **Placebo** | 18.93 ± 1.74^a^ | 18.85 ± 1.59^a^ | 23.42 ± 1.95^a^ | 22.27 ± 1.56^a^ | F_3,116_=1.84, P1=0.143 |
|  | **Synbiotics** | 18.98 ± 1.59^c^ | 11.38 ± 1.05^b*^ | 6.91 ± 0.40^a*^ | 4.79 ± 0.24^a*^ | F_3,116_=40.97, P1=0.000 |
|  |  | P1=0.984 | P1=0.000 | P1=0.000 | P1=0.000 |  |
|  | **Probiotic** | 18.14 ± 1.56^c^ | 12.42 ± 1.22^b*^ | 9.07 ± 0.75^a*^ | 7.67 ± 0.58^a*#^ | F_3,116_=18.02, P1=0.000 |
|  |  | P1=0.733, P2=0.718 | P1=0.001, P2=0.578 | P1=0.000, P2=0.218 | P1=0.000, P2=0.039 |  |
|  | **Treatment** | F_2,87_=0.083, P2=0.92 | F_2,87_=9.58, P2=0.000 | F_2,87_=53.45, P2=0.000 | F_2,87_=93.06, P2=0.000 |  |

*: a significant difference (P1<0.05), as compared to the control group.

#: a significant difference (P2<0.05), as compared to the symbiotic group.

**Table 5.** **Postoperative short-term outcome**s

|  | **Placebo cases**  **(n=30)** | **Probiotic cases**  **(n=30)** | **Synbiotic cases**  **(n=30)** | ***P-Value*** |
| --- | --- | --- | --- | --- |
| **Bleeding (%)** | 1/30 (3.3%) | 1/30 (3.3%) | 1/30 (3.3%) | 1.000 |
| **Mean Day to First Stool (day)** | 5.00 ± 0.13 | 4.00 ± 0.09* | 4.00 ± 0.14* | *0.000* |
| **Post-operative stay (day)** | 10.00 ± 0.13 | 10.00 ± 0.14 | 10.00 ± 0.14 | 1.000 |
| **Return to usual activity (day)** | 14.00 ± 0.13 | 14.00 ± 0.17 | 14.00 ± 0.08 | 1.000 |
| **Incidence of infectious complications (%)** |  |  |  |  |
| Bacteremia | 15/30 (50.0%) | 7/30 (23.3%) | 4/30 (13.3%)* | 0.047 |
| Wound infection | 2/30 (6.7%) | 1/30 (3.3%) | 1/30 (3.3%) | 0.770 |
| Pneumonia | 4/30 (13.3%) | 2/30 (6.7%) | 2/30 (6.7%) | 0.578 |
| Urinary tract infection | 5/30 (16.7%) | 5/30 (16.7%) | 4/30 (13.3%) | 0.919 |
| **Incidence of non-infectious complications (%)** |  |  |  |  |
| Anastomotic Leakage | 3/30 (10.0%) | 0/30 (0 %)* | 0/30 (0 %)* | 0.032 |
| Diarrhea | 17/30 (56.7%) | 10/30 (33.3%)* | 8/30 (26.7%)* | *0.044* |
| Abdominal distension | 16/30 (53.3%) | 10/30 (33.3%)* | 7/30 (23.3%)* | 0.042 |
| **Mortality (n)** | N/P (0 %) | N/P (0%) | N/P (0%) | 0.364 |

Data are presented as mean ± standard error and number (percentages). P<0.05: Significant differences according to Chi-squared and One-way ANOVA.
